# Tolerance and growth in children with cow’s milk allergy fed a thickened extensively hydrolyzed casein-based formula

**DOI:** 10.1186/s12887-016-0637-3

**Published:** 2016-07-18

**Authors:** Christophe Dupont, Elena Bradatan, Pascale Soulaines, Rita Nocerino, Roberto Berni-Canani

**Affiliations:** Pediatric Gastroenterology, Hepatology and Nutrition Department, Necker Children’s Hospital, 149, rue de Sèvres, 75015 Paris, France; Department of Pediatrics, Regional Hospital, Namur, Belgium; Department of Translational Medical Science and European Laboratory for the Investigation of Food Induced Diseases and CEINGE Advanced Biotechnologies, University of Naples “Federico II”, Naples, Italy

**Keywords:** Cow’s milk allergy, Cow’s Milk-related Symptom Score (CoMiSS), Hypoallergenic extensively hydrolyzed casein-based formula, Infant growth, Scoring Atopic Dermatitis (SCORAD)

## Abstract

**Background:**

In case of cow’s milk allergy (CMA), pediatric guidelines recommend for children the use of extensively hydrolyzed formulas (eHFs) as elimination diet. According to the American Academy of Pediatrics, the hypoallergenicity of each specific eHF should be tested in subjects with CMA.

**Methods:**

A prospective, multicenter trial was performed to assess the tolerance/hypoallergenicity of a thickened casein-based eHF (eHCF, Allernova AR®, United Pharmaceuticals, France) in infants aged <12 months with CMA proven by a double-blind placebo-controlled food challenge. Its efficacy, measured through allergy symptoms monitoring and Cow’s Milk-related Symptom Score (CoMiSS) calculation, and safety were evaluated during a 4-month feeding period. Growth z-scores were computed based on WHO anthropometric data.

**Results:**

Thirty infants (mean age: 4.8 ± 3.0 months) with CMA proven by a DBPCFC tolerated the eHCF during the 4-month study. The CoMiSS, crying and regurgitation scores significantly decreased by 4.2 ± 4.0, 0.9 ±1.2 and 0.7 ± 1.1 respectively, after 14 days of feeding (*p <* 0.001). The Scoring Atopic Dermatitis index, of 33.2 ± 14.8 at inclusion in 9 patients, significantly decreased by 15.5 ± 6.7 and 21.1 ± 11.2, after 14 and 45 days of feeding, respectively (*p <* 0.001). The percentage of infants having normal stool consistency (soft or formed stools) significantly improved from 66.7 % (20/30) at inclusion to 90.0 % (27/30) after 14 days of feeding (*p =* 0.020). The growth z-scores, negative at study inclusion, significantly improved over the 4-month study. No adverse event was related to the eHCF.

**Conclusion:**

The thickened eHCF was tolerated by more than 90 % of included allergic infants with 95 % confidence interval and can therefore be considered as hypoallergenic in accordance with current guidelines. The improvement of growth indices and absence of related adverse events confirmed its safety. Results of this trial back the use of the tested thickened eHCF as an efficient and safe alternative in children with CMA.

**Trial registration:**

ClinicalTrials.gov, number NCT02351531, registered on 27 January 2015

**Electronic supplementary material:**

The online version of this article (doi:10.1186/s12887-016-0637-3) contains supplementary material, which is available to authorized users.

## Background

Cow’s milk allergy (CMA) is an immune-mediated reaction which can either be antibody-driven (IgE-mediated) or cell-mediated (non-IgE-mediated) or mixed, and elicits reactions which are reproducible upon re-exposure to cow’s milk proteins (CMP) [1]. Estimates of CMA prevalence depend on the diagnosis procedure used; recently, a meta-analysis stated an overall pooled estimate for 0–1 year old infants of point prevalence of CMA reported by parents of 4.2 % (95 % confidence interval (CI): 3.2–5.4), decreasing to 2.0 % (1.5–2.5) when CMA was proven with a double-blind placebo-controlled food challenge (DBPCFC) [2]. CMA manifests through diverse and non-specific symptoms, rendering the CMA diagnosis very difficult [3–5]. CMA symptoms mainly concern the cutaneous area, the respiratory and gastrointestinal tracts but can also be general [3–6]. The DBPCFC is therefore considered as the gold standard for CMA diagnosis [4, 6, 7]. CMA treatment consists in the elimination of any source of non-hydrolyzed CMP from the diet, which is mainly achieved in children by using extensively hydrolyzed formulae (eHFs) based on cow’s milk [4–6, 8]. As the molecular weight profile of a given hydrolysate cannot predict potential reaction in a given child [9], the American Academy of Pediatrics recommended that tolerance/hypoallergenicity of any formula intended for allergic children should be clinically tested in that specific population [10]. eHFs should also be tested for their growth adequacy in allergic children [6, 8, 11] as CMA may result in growth retardation [12]. Regurgitations, which are the most typical presentation of infantile gastro-esophageal reflux, are common complaints in infancy [13]. Although they may be a symptom of CMA, they may also occur in allergic infants independently of their allergic disease. To effectively manage both conditions in infants, the new eHF based on casein (eHCF) tested in this trial has been thickened. Therefore, this trial was aimed at evaluating the tolerance/hypoallergenicity of the thickened eHCF in infants with CMA proven through DBPCFC, as well as its efficacy on allergy symptoms and its impact on growth during a 4-month feeding period.

## Methods

### Study population

Infants aged between 1 and 12 months with CMA, either confirmed through a DBPCFC within 3 months prior to inclusion, or highly suspected based on specific suggestive symptoms, were included in this prospective, multicenter study. The main exclusion criteria were: infants mainly or exclusively breastfed with mother’s willingness to continue breastfeeding, infants who would need an amino acid-based formula (AAF) according to pediatric recommendations [3, 4], infants fed an eHF with no improvement of their allergy symptoms, infants who refused to drink an eHF any time prior to inclusion and infants fed the non-thickened version of the tested formula. At study enrolment, if CMA was not already diagnosed by a DBPCFC, such a challenge had to be performed within the 3 months following inclusion. In case of negative challenge, subject’s participation in the trial ended and the patient was included in the Safety population (defined in *Study outcomes*) only. The challenge was performed according to guidelines [3]: in short, the child was fed on two different days with volumes being increased every 20 min under medical supervision of either an AAF (Neocate®, Nutricia, Germany) as placebo or a formula which blended two thirds of a standard CMP-based infant formula with one third of Neocate® to ensure double-blinding. The child was observed for 2 additional hours after the last dosage administration to monitor immediate reactions. After completion of both challenge days, in the absence of immediate reaction to CMP, the child had to drink at least 250 ml per day of a standard CMP-based formula for up to one week [3]. At home, parents monitored the appearance of delayed allergy reactions and reported them to the physician. In case of delayed allergy reaction, the exclusive bottle-feeding of the tested formula was immediately reinitiated. If no reaction occurred either during both challenge test days or during one-week feeding with the standard CMP-based formula, cow’s milk challenge was considered negative and CMA diagnosis was excluded.

### Study formula feeding

Infants were exclusively bottle-fed the tested formula (Allernova AR®, Novalac, United Pharmaceuticals, France) for 4 months. The tested formula contains an extensively casein-based hydrolysate as protein source and is thickened with a patented complex containing fibers (0.5 g/100 ml), mainly composed of pectin, to reduce regurgitation but also to help intestinal transit regulation. Its nutritional composition complied with the applicable European regulation, particularly regarding the amino acid profile.

### Study outcomes

The primary outcome was the tolerance/hypoallergenicity of the tested formula, defined as the absence, in infants with a proven CMA, of any allergy symptoms that led to study discontinuation during the first two weeks. It was evaluated on patients in the Tolerance/Hypoallergenicity population, i.e. all patients fed the tested formula at least once and for whom the CMA was proven. Patients fed the tested formula at least once formed the Safety population. The secondary outcomes were the efficacy of the studied formula on allergy symptoms (mainly including the evolution of the Cow’s Milk-related Symptom Score [14] and the main CMA symptom), its impact on growth parameters and on parents and investigators satisfaction. These outcomes were assessed on the Full Analysis Set (FAS) population comprising patients from the Safety population with evaluation of the main efficacy criterion at baseline and at 2 weeks. Adverse events (AEs) were registered in patients in the Safety population.

### Study interventions

Visits were planned 14, 45, 90 and 120 days after inclusion. Other CMA diagnosis tests, dosage of serum IgE specific to cow’s milk (sIgE), skin prick test (SPT) and atopy patch test (APT), were performed if deemed necessary by the physician according to his usual practice; when carried out before study inclusion, the results of these tests were also collected. From serum, sIgE were analyzed with enzymatic immunoassay (Phadia 100 ThermoFisher Scientific CAP system), the limit of detection being 0.1 kU/l. For SPT, commercial UHT milk, and histamine dihydrochloride (10 mg/ml) and isotonic saline solution (NaCl 0.9 %) as positive and negative control, respectively, were applied to the patients’ volar forearm. SPT were performed using a 1-mm single peak lancet (ALK, Copenhagen, Denmark) in Italy and Stallerpoint® (Stallergenes SA, France) in France and Belgium. Reactions were recorded on the basis of the largest diameter (in millimeters) of the wheal and flare at 15 min. The SPT result was considered “positive” if the wheal diameter induced by cow’s milk minus that induced by negative control was larger than 3 mm. For APT, 1–2 drops of commercial UHT milk was placed on filter paper and applied with adhesive tape to the unaffected skin of the child’s back, using 12-mm aluminum cups (Finn Chambers® on Scan pore). Isotonic saline solution was the negative control. The occlusion time was 48 h and results were read 20 min and 24 h after removal of the cups. The test result was considered positive if at least a significant erythema was present. IgE-mediated was defined as having either positive sIgE or positive SPT to cow’s milk.

Parents were instructed to eliminate any milk or dairy products from the diet throughout the entire study and to not introduce hen’s egg, soy protein, peanut or any new food in their infant’s diet in the first two weeks of the study. Patient selection was performed in hospital outpatient clinics and private practices in France, Belgium and Italy.

### Study measurements

During 3 days before each visit, parents were asked to record data on formula intake, number of regurgitations, stool patterns and duration of crying. At inclusion and each follow-up visit, the presence and severity of CMA symptoms were registered by the same investigator, based on clinical examination and parents report. CMA symptoms were itemized for each concerned area: cutaneous (urticaria, angioedema and eczema, the severity of the latter being assessed as mild, moderate or severe, on head, neck and trunk and on arms, hands, legs and feet), respiratory symptoms (such as wheezing, rhinitis, bronchitis, bronchospasm, their severity being assessed as slight, mild or severe), digestive (regurgitations assessed through the regurgitation scale defined by Vandenplas et al. [15], vomiting, bloody stools, stool consistency assessed through the Bristol stool scale [16]), and digestive discomfort as general symptom (mild, moderate or severe intensity and reflected by abdominal pain, gas, bloating and irritability). Daily unexplained crying time was registered through a scale with the following points: less than one hour/day, 1–1.5 h/d, 1.5–2 h/d, 2–3 h/d, 3–4 h/d, 4–5 h/d and more than 5 h/d. During a workshop held in 2014, a Cow’s Milk-related Symptom Score (CoMiSS) was defined [14]. It comprises five items (crying, regurgitations, stool consistency, skin and respiratory symptoms), which were all assessed during the study, enabling the calculation of the CoMiSS for each infant at each visit. Eczema severity was assessed using the Scoring Atopic Dermatitis index (SCORAD) [17], as this score is a valid tool, commonly and easily used by hospital physicians. Because of the diversity of CMA symptoms in general [3–5], the pediatrician had to determine the main CMA symptom for each subject at baseline, and assess its evolution at each follow-up visit. At each visit, the pediatrician measured weight, length and head circumference and registered stool frequency, sleep quality (either agitated, i.e. excessive waking with no clear cause, or quiet, i.e. absence of or few awakenings) and adverse events (AEs).

### Statistical analysis

In order to be considered hypoallergenic, a formula must demonstrate that with 95 % CI, it does not provoke allergic reactions in 90 % of subjects with confirmed CMA [10]. In case of no reaction, a sample size of 29 participants is sufficient.

For quantitative parameters, intra-group changes were analysed using the Student’s test or Wilcoxon’s test (non-normal data). For qualitative parameters, changes from baseline within treatment group were analysed by symmetry test, or by McNemar test for binary variables. Statistical analyses were conducted using SAS version 9.2 (SAS Institute Inc., United States). Significance was set at *p <* 0.05. Weight-for-age (WFA), length-for-age (LFA), weight-for-length (WFL), body mass index (BMI)-for-age and head circumference-for-age (HCA) z-scores were computed based on WHO anthropometric data [18]. The CoMiSS was calculated for each patient and at each visit [14].

The study design was approved by independent ethic committees: Ile-de-France III (Paris, France), Medical Ethics Committee of the Regional Hospital of Namur (Belgium) and Ethics Committee of the University of Naples, Federico II. This study was conducted in accordance with ethical standards laid down by the Declaration of Helsinki. Parents, or others legally responsible for the infants, provided written consent regarding their acceptance to participate and the study procedures.

## Results

**Fig. 1:**
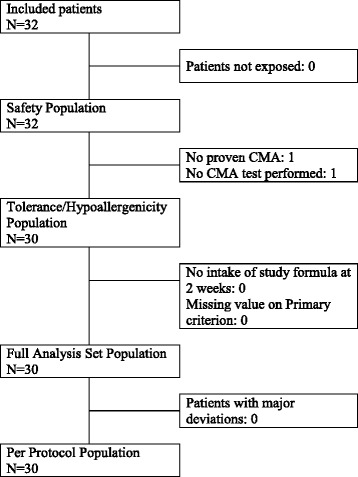
Study flow chart. N: number of subjects

Thirty two infants were included in 3 centers from November 2013 to July 2014. CMA was confirmed in 30 of them through a DBPCFC and therefore constituted the hypoallergenicity population (Fig. 1). One infant tolerated a cow’s milk-based formula introduced by his parents 5 days after study inclusion, excluding the CMA diagnosis. Another infant dropped out of the study before CMA could be confirmed because of his parents’ wish to withdraw. According to the investigator, it was not due to any medical reason, and the patient could have continued to participate in the study. All those 30 infants completed the 4-month study.

Main baseline characteristics of included subjects are described in Table 1 and Additional file 1 [19, 20]. 70.0 % (21/30) of infants had IgE-mediated CMA. At inclusion, 80.0 % of infants (24/30) were on elimination diet. DBPCFC was performed for 16.7 % of patients at a median of 3.9 [range: 0.1-8.7] weeks before study inclusion, and during study course for 83.3 % of infants, at a median of 0.4 [0.1-11.3] weeks after study inclusion. 22 patients had immediate reactions to CMP during the DBPCFC (Table 2).

**Table 1:** Main demographic and clinical characteristics of FAS population (*N =* 30) at inclusion

Characteristics	
Boys, N (%)	18 (60.0)
Age, mean (± SD), months	4.8 (3.0)
Gestational age, mean (± SD), weeks	38.7 (1.0)
WFA z-score at birth, mean (± SD)	−0.1 (1.1)
LFA z-score at birth, mean (± SD)	0.0 (1.3)
*Feeding history*	
Ever breastfed, N (%)	25 (83.3)
Duration of exclusive breastfeeding, mean (± SD), weeks	11.5 (7.7)
Duration of partial breastfeeding, mean (± SD), weeks	8.5 (6.1)
Type of feeding at study entry, N (%)	
Exclusively formula-fed	29 (96.7)
Partially breast-fed^a^	1 (3.3)
Type of formula used before study inclusion, N (duration of use, mean ± SD, weeks)	
Non-hydrolyzed CMP-based formula	6 (8.5 ± 5.0)
Extensively hydrolyzed formula based on CMP	13 (6.2 ± 8.8)
Amino acid-based formula	6 (2.7 ± 1.1)
Vegetable-based formula	5 (2.6 ± 2.1)
*Anthropometric data*	
WFA z-score, mean (± SD)	−0.8 (0.8)
LFA z-score, mean (± SD)	−0.7 (1.0)
WFL z-score, mean (± SD)	−0.4 (1.1)
BMI-for-age z-score, mean (± SD)	−0.6 (1.0)
HCA z-score, mean (± SD)	−0.3 (1.2)
*Allergy characteristics*	
Family history of allergy‡, N (%)	10 (33.3)
Age at onset of allergy symptoms, mean (± SD), months	2.5 (2.3)
Time since the start of the exclusion diet, median [min – max; IQR], weeks	2.7 [0.0–36.0; 1.1–6.3]
Delay between onset of allergy symptoms and start of exclusion diet, median [min – max; IQR], weeks	1.3 [0.0–34.9; 0.6–4.1]
Types of first allergy symptoms, N (%)	
Exclusively digestive	17 (56.7)
Exclusively cutaneous	10 (33.3)
At least two concerned areas	3 (10.0)
CMA diagnosis tests, number of subjects with positive reactions/number of subjects with test performed (%)	
Atopy patch test to CMP	8/18 (44.4)
Skin prick test to CMP	20/30 (66.7)
Serum IgE specific to CMP	3/6 (50.0)

*N* number of subjects, *min* minimum, *max* maximum, *IQR* interquartile range

^a^the mother excluded CMP from her regimen; ‡at least one parent or sibling with confirmed allergy

**Table 2:** Characteristics related to DBPCFC of the FAS population (*N =* 30)

Characteristics	
Immediate reactions to CMP, N (%)	22 (73.3)
Types of immediate reactions to CMP, N (%)	
Digestive signs	19 (86.4)
Local cutaneous signs	7 (31.8)
General cutaneous signs	6 (27.3)
Laryngeal edema	2 (9.1)
Bronchospasm	1 (4.5)
Delayed reactions to CMP, N (%)	10 (33.3)
Types of delayed reactions to CMP, N (%)	
Digestive	8 (80.0)
Cutaneous	5 (50.0)
Cumulative dose of non-hydrolyzed CMP-based formula eliciting immediate reactions, median [minimum-maximum], ml	15 [5–95]
Time for eliciting immediate reactions, mean (± SD), minutes	83.8 (16.1)

*N* number of subjects

No infant from the Tolerance/Hypoallergenicity population dropped out of the study and all of them tolerated the tested formula.

The main CMA symptom was digestive for 63.3 % (19/30) of infants, cutaneous for 33.3 % (10/30) of infants and general for one infant. It was resolved or improved as of day 14 for 83.3 % of the patients (*p <* 0.001, proportion test) and for 100 % of patients within 45 days. The mean (± standard deviation (SD)) CoMiSS, regurgitation and crying scores significantly decreased by 4.2 (±4.0), 0.7 (±1.1) and 0.9 (±1.2) respectively after 14 days of feeding (Table 3). At inclusion, 90.0 % (27/30) of infants cried ≥1.5 h per day, significantly decreasing to 66.7 and 46.7 % after 14 and 45 days respectively (*p* = 0.020; *p <* 0.001, McNemar test). At inclusion, 3 patients had angioedema, this symptom disappearing after 14 days. 9 patients had eczema at inclusion with a mean (±SD) SCORAD index of 33.2 (±14.8) which significantly decreased by 15.5 (±6.7) and 21.1 (±11.2) after 14 and 45 days, respectively (*p* < 0.001, Student’s test). At inclusion, 22 infants experienced vomiting; at 14 days, this number was significantly reduced by half (*p* = 0.002, McNemar test). 6 patients had bloody stools at inclusion, decreasing to 3 after 14 days, and to none after 45. Normal stool consistency (formed or soft stools), present in 66.7 % (20/30) of infants at inclusion, significantly increased to 90.0 % (27/30) after 14 days (*p* = 0.020, McNemar test).

**Table 3:** Change from baseline of CoMiSS and parameters contributing to the CoMiSS at 14 days

	Inclusion (*N =* 30)	D14 (*N =* 30)
CoMiSS, mean (± SD)	7.4 (4.4)	3.2 (2.3)*
Regurgitation score^a^, mean (± SD)	1.6 (1.6)	0.9 (1.0)*
Crying score^a^, mean (± SD)	1.7 (1.1)	0.8 (0.6)*
Stool consistency, N (%)		
Type I/II (hard)	6 (20.0)	2 (6.7)
Type III/IV (formed)	16 (53.3)	20 (66.7)
Type V (soft)	4 (13.3)	7 (23.3)
Type VI (mushy)	3 (10.0)	1 (3.3)
Type VII (watery)	1 (3.3)	0 (0.0)
Urticaria, N (%)		
Presence	7 (23.3)	0 (0.0)
Absence	23 (76.7)	30 (100.0)
Eczema, N (%)		
Head, neck, trunk		
Absence	21 (70.0)	24 (80.0)
Mild	3 (10.0)	4 (13.3)
Moderate	5 (16.7)	2 (6.7)
Severe	1 (3.3)	0 (0.0)
Arms, hands, legs, feet		
Absence	23 (76.7)	24 (80.0)
Mild	3 (10.0)	3 (10.0)
Moderate	3 (10.0)	3 (10.0)
Severe	1 (3.3)	0 (0.0)
Respiratory symptoms, N (%)		
Absence	25 (83.3)	28 (93.3)
Mild	4 (13.3)	1 (3.3)
Moderate	1 (3.3)	1 (3.3)

*D* day, *N* number of subjects

*P-values vs. inclusion < 0.001 (Wilcoxon’s test)

^a^Sub-scores included in the calculation of the CoMiSS

Digestive discomfort, present in 25 patients at inclusion, of which 12 patients had symptoms of moderate/severe intensity, decreased to 17 patients after 14 days (*p* = 0.011, McNemar test), of which only one patient had symptoms of moderate/severe intensity. Stool frequency did not significantly change after 14 and 45 days. 73.3 % (22/30) of infants had 1–3 stools/day on day 14. Agitated sleep significantly decreased from 83.3 % (25/30) of infants at baseline to 43.3 % (13/30) after 14 days (*p* = 0.001, McNemar test).

The mean (±SD) feeding duration was 113.6 (±27.8) days and the mean daily intake of study formula was higher than 600 ml/day during the entire study course. 33 AEs were reported in 24 patients: 48.5 % (16/33) were respiratory infections and one third gastroenteritis. None were related to the tested formula nor led to feeding discontinuation of the tested formula. No serious AEs were reported. Between birth and inclusion, the mean (±SD) WFA and LFA z-scores had significantly decreased by 0.7 (±1.0) and 0.6 (±1.1), respectively (*p* < 0.001; *p* = 0.003, Student’s test). All growth indices, negative at study inclusion, showed significant improvements within the 4-month study (Table 4). As of 14 days of feeding, 73.3 % (22/30) of the investigators and 71.4 % (20/28) of the parents were globally satisfied with the formula, 75.8 % (22/29) of parents being satisfied or very satisfied in particular with their child’s acceptance of the formula’s taste.

**Table 4:** Growth indices at inclusion and follow-up visits (D45, D90 and D120)

	Inclusion	D45	D90	D120
Age, mean (± SD), months	4.8 (3.0)	6.3 (3.1)	7.8 (3.0)	8.7 (3.0)
Weight-for-age z-score, mean (± SD)	−0.8 (0.8)	−0.2 (0.7)	0.1 (0.7)	0.4 (0.8)
N		29	29	29
P-values vs. baseline		<0.001^a^	<0.001^a^	<0.001^a^
Length-for-age z-score, mean (± SD)	−0.7 (1.0)	−0.3 (1.2)	0.0 (1.2)	0.4 (1.1)
N		29	29	28
P-values vs. baseline		0.008^a^	<0.001^a^	<0.001^a^
Weight-for-length z-score, mean (± SD)	−0.4 (1.1)	0.0 (0.8)	0.2 (0.6)	0.3 (0.7)
N		29	29	28
P-values vs. baseline		0.002^a^	<0.001^a^	<0.001^a^
Body mass index-for-age z-score, mean (± SD)	−0.6 (1.0)	−0.1 (0.8)	0.2 (0.7)	0.3 (0.8)
N		29	29	28
P-values vs. baseline		0.001^a^	<0.001^a^	<0.001^a^
Head circumference-for-age z-score, mean (± SD)	−0.3 (1.2)	0.2 (1.0)	0.7 (0.8)	1.1 (0.9)
N		27	29	29
P-values vs. baseline		<0.001^b^	<0.001^b^	<0.001^b^

*D* day, *N* number of subjects

^a^Student’s test

^b^Wilcoxon’s test

## Discussion

This study demonstrates the hypoallergenicity, efficacy and positive effect on growth catch-up of the studied eHCF in infants with CMA. As all infants with CMA, confirmed by a DBPCFC, tolerated the tested formula, this formula meets the hypoallergenicity criteria of the American Academy of Pediatrics [10].

In this study, CMA was proven in all subjects by a DBPCFC, the gold standard for CMA diagnosis [3, 4, 7]. In addition, in the absence of a reference group, which allows controlling for the natural evolution of the disease, the symptom evolution was first evaluated 2 weeks after study enrollment, which is close enough to the time of diagnosis to exclude the possibility of a natural evolution of symptoms [3, 4].

The efficacy of the studied eHCF was thoroughly documented in this trial, by assessing all parameters contributing to an existing Symptoms-Based Score (SBS) [21–23]. A working group recently considered the SBS as a valuable tool for evaluating and quantifying the evolution of CMA symptoms during therapeutic interventions and renamed it Cow’s Milk-related Symptom Score (CoMiSS) [14]. Here, this score was significantly reduced as early as 14 days after eHCF feeding initiation. A similar evolution was reported in previous studies following young infants with proven CMA and under elimination diet by using this score. In 37 and 34 infants fed respectively an eHF based on rice proteins and an eHCF, the mean SBS (±SD) significantly decreased after one month-feeding from 13.0 (±5.2) to 3.5 (±2.3) and from 14.3 (±3.3) to 5.7 (±3.7) [22, 23]. In another study, 59 infants fed an eHCF or an eHF based on whey proteins (eHWF) showed a mean SBS of 13.6 (±1.7) at inclusion that decreased to 5.1 (±3.4) after one month-feeding [21]. Compared with these previous results, the mean CoMiSS value at inclusion reported here was relatively small and lower than the value (≥12) which could have an 80 % positive predictive value for CMA diagnosis at the start of an elimination diet followed by a decrease to ≤6 under an elimination diet with eHF. This can be explained by the fact that 80.0 % of enrolled infants were on an elimination diet, more than half with eHF based on CMP (54.2 %), one quarter with AAF and 20.8 % with vegetable-based formulas.

In the absence of a validated CMA severity score [14], previous similar studies frequently focused on the SCORAD index evolution, a validated tool for assessment of eczema severity [17], especially since some eHFs, but not all [24], based on casein [25, 26] or whey proteins [25, 27] efficiently induced a decrease in this score in CMA patients. In this present study, less than one third of patients had eczema at inclusion, and their SCORAD index significantly decreased 14 and 45 days after eHCF feeding initiation.

CMA treatment relies on dietary elimination of intact CMP [3, 4, 8] which may induce nutritional deficiencies in children in case of an inadequate elimination diet. As shown by negative growth indices in children with CMA at study enrollment [26], CMA is frequently associated with a growth deficit [28, 29]. The mechanisms for impaired growth are not entirely clear but may rise from a sustained inflammation and subsequent reduced bioavailability or loss of nutrients in the gastrointestinal tract, while metabolic requirements may be increased by skin inflammation and disrupted sleep [12]. A delayed diagnosis and thus a delay in initiation of an appropriate dietary management is a risk factor for impaired growth in children with a food allergy [30]. Here, CMA symptoms appeared during the first months of life, as previously reported [5, 6], and the median [range] delay between their appearance and implementation of an elimination diet was 1.3 [0.0–34.9] weeks. As shown before [26, 31, 32], WFA and LFA z-scores significantly decreased between birth and study inclusion. Feeding with the study eHCF enabled growth normalization in line with WHO standards, as already observed for eHCF feeding [23, 26, 32].

In this study, whatever their CMA type, IgE-mediated or not, all infants tolerated the eHCF during 4 months, and notably with consumptions of high volumes. Parents sometimes ask for an eHF feeding change for various reasons, for example because of a poor taste acceptability—eHFs are known for their bitterness [3, 4, 9, 33, 34]—or for poor digestive comfort including regurgitations [35]. All infants who were already on an elimination diet for various time periods and with different types of formulas devoid of non-hydrolyzed CMP tolerated the studied eHCF.

## Conclusions

The tested thickened eHCF was tolerated during 4 months by all infants with CMA proven by a DBPCFC, either IgE or non-IgE mediated and whether already fed or not an elimination diet. The formula feeding efficiently reduced the SCORAD index in patients with eczema and the CoMiSS, a recently developed tool to follow allergy symptoms, in all subjects. This study was adequately powered to demonstrate the hypoallergenicity of the studied formula, but the results observed on allergy symptoms and growth indices deserve confirmation in a larger sample.

The CONSORT guidelines [36], when applicable, were followed for reporting data of this study.

## Abbreviations

AAF, amino acid-based formula; AEs, adverse events; APT, atopy patch test; BMI, body mass index; CI, confidence interval; CMA, cow’s milk allergy; CMP, cow’s milk proteins; CoMiSS, Cow’s Milk-related Symptom Score; DBPCFC, double-blind placebo-controlled food challenge; eHCF, extensively hydrolyzed casein-based formula; eHFs, extensively hydrolyzed formulae; eHWF, extensively hydrolyzed whey-based formula; FAS, full analysis set; HCA, head circumference-for-age; LFA, length-for-age; SBS, Symptoms-Based Score; SCORAD, scoring atopic dermatitis; SD, standard deviation; SPT, skin prick test; WFA, weight-for-age; WFL, weight-for-length

## Additional file

Additional file 1: Table S1. Supplementary baseline characteristics of FAS population (*N =* 30) at inclusion. (DOCX 16 kb)

## References

[1] Johansson Bieber T, Dahl R, Friedmann PS, Lanier BQ, Lockey RF (2004). Revised nomenclature for allergy for global use: Report of the Nomenclature Review Committee of the World Allergy Organization, October 2003. J Allergy Clin Immunol.

[2] Nwaru BI, Hickstein L, Panesar SS, Roberts G, Muraro A, Sheikh A (2014). Prevalence of common food allergies in Europe: a systematic review and meta-analysis. Allergy.

[3] Vandenplas Y, Koletzko S, Isolauri E, Hill D, Oranje AP, Brueton M (2007). Guidelines for the diagnosis and management of cow’s milk protein allergy in infants. Arch Dis Child.

[4] Koletzko S, Niggemann B, Arato A, Dias JA, Heuschkel R, Husby S (2012). European Society of Pediatric Gastroenterology, Hepatology, and Nutrition. Diagnostic approach and management of cow’s-milk protein allergy in infants and children: ESPGHAN GI Committee practical guidelines. J Pediatr Gastroenterol Nutr.

[5] Luyt D, Ball H, Makwana N, Green MR, Bravin K, Nasser SM (2014). BSACI guideline for the diagnosis and management of cow’s milk allergy. Clin Exp Allergy.

[6] Fiocchi A, Brozek J, Schünemann H, Bahna SL, von Berg A, Beyer K (2010). World Allergy Organization (WAO) Diagnosis and Rationale for Action against Cow’s Milk Allergy (DRACMA) Guidelines. Pediatr Allergy Immunol.

[7] Dupont C (2014). Diagnosis of cow’s milk allergy in children: determining the gold standard?. Expert Rev Clin Immunol.

[8] Dupont C, Chouraqui JP, de Boissieu D, Bocquet A, Bresson JL, Briend A (2012). Dietary treatment of cows’ milk protein allergy in childhood: a commentary by the Committee on Nutrition of the French Society of Paediatrics. Br J Nutr.

[9] Høst A, Koletzko B, Dreborg S, Muraro A, Wahn U, Aggett P (1999). Dietary products used in infants for treatment and prevention of food allergy. Joint Statement of the European Society for Paediatric Allergology and Clinical Immunology (ESPACI) Committee on Hypoallergenic Formulas and the European Society for Paediatric Gastroenterology, Hepatology and Nutrition (ESPGHAN) Committee on Nutrition. Arch Dis Child.

[10] American Academy of Pediatrics (2000). Committee on Nutrition. Hypoallergenic infant formulas. Pediatrics.

[11] Giovannini M, D’Auria E, Caffarelli C, Verduci E, Barberi S, Indinnimeo L (2014). Nutritional management and follow up of infants and children with food allergy: Italian Society of Pediatric Nutrition/Italian Society of Pediatric Allergy and Immunology Task Force Position Statement. Ital J Pediatr.

[12] Meyer R, Venter C, Fox AT, Shah N (2012). Practical dietary management of protein energy malnutrition in young children with cow’s milk protein allergy. Pediatr Allergy Immunol.

[13] Nelson SP, Chen EH, Syniar GM, Christoffel KK (1997). Prevalence of symptoms of gastroesophageal reflux during infancy. A pediatric practice-based survey. Pediatric Practice Research Group. Arch Pediatr Adolesc Med.

[14] Vandenplas Y, Dupont C, Eigenmann P, Host A, Kuitunen M, Ribes-Koninckx C (2015). A workshop report on the development of the Cow’s Milk-related Symptom Score awareness tool for young children. Acta Paediatr.

[15] Vandenplas Y, Hachimi-Idrissi S, Casteels A, Mahler T, Loeb H (1994). A clinical trial with an “anti-regurgitation” formula. Eur J Pediatr.

[16] Lewis SJ, Heaton KW (1997). Stool form scale as a useful guide to intestinal transit time. Scand J Gastroenterol.

[17] Consensus Report of the European Task Force on Atopic Dermatitis (1993). Severity scoring of atopic dermatitis: the SCORAD index. Dermatology.

[18] WHO Multicentre Growth Reference Study Group (2006). WHO Child Growth Standards: Length/height-for-age, weight-for-age, weight-for-length, weight-for-height and body mass index-for-age: Methods and development.

[19] Aggett P, Agostoni C, Axelsson I, Goulet O, Hernell O, Koletzko B (2003). Core data for nutrition trials in infants: a discussion document--a commentary by the ESPGHAN Committee on Nutrition. J Pediatr Gastroenterol Nutr.

[20] Koletzko B, Fewtrell M, Gibson R, van Goudoever JB, Hernell O, Shamir R (2015). Core data necessary for reporting clinical trials on nutrition in infancy. Ann Nutr Metab.

[21] Vandenplas Y, Steenhout P, Grathwohl D, Althera Study Group (2014). A pilot study on the application of a symptom-based score for the diagnosis of cow’s milk protein allergy. SAGE Open Medicine..

[22] Vandenplas Y, De Greef E, Hauser B, Paradice Study Group (2014). An extensively hydrolysed rice protein-based formula in the management of infants with cow’s milk protein allergy: preliminary results after 1 month. Arch Dis Child.

[23] Vandenplas Y, De Greef E, ALLAR study group (2014). Extensive protein hydrolysate formula effectively reduces regurgitation in infants with positive and negative challenge tests for cow’s milk allergy. Acta Paediatr.

[24] Niggemann B, Binder C, Dupont C, Hadji S, Arvola T, Isolauri E (2001). Prospective, controlled, multi-center study on the effect of an amino-acid-based formula in infants with cow’s milk allergy/intolerance and atopic dermatitis. Pediatr Allergy Immunol.

[25] Niggemann B, von Berg A, Bollrath C, Berdel D, Schauer U, Rieger C (2008). Safety and efficacy of a new extensively hydrolyzed formula for infants with cow’s milk protein allergy. Pediatr Allergy Immunol.

[26] Dupont C, Hol J, Nieuwenhuis EE (2015). Cow’s Milk Allergy Modified by Elimination and Lactobacilli study group. An extensively hydrolysed casein-based formula for infants with cows’ milk protein allergy: tolerance/hypo-allergenicity and growth catch-up. Br J Nutr.

[27] Isolauri E, Sütas Y, Mäkinen-Kiljunen S, Oja SS, Isosomppi R, Turjanmaa K (1995). Efficacy and safety of hydrolyzed cow milk and amino acid-derived formulas in infants with cow milk allergy. J Pediatr.

[28] Isolauri E, Sütas Y, Salo MK, Isosomppi R, Kaila M (1998). Elimination diet in cow’s milk allergy: risk for impaired growth in young children. J Pediatr.

[29] Agostoni C, Grandi F, Scaglioni S, Giannì ML, Torcoletti M, Radaelli G (2000). Growth pattern of breastfed and nonbreastfed infants with atopic dermatitis in the first year of life. Pediatrics.

[30] Venter C, Laitinen K, Vlieg-Boerstra B (2012). Nutritional aspects in diagnosis and management of food hypersensitivity-the dietitians role. J Allergy (Cairo).

[31] Savino F, Castagno E, Monti G, Serraino P, Peltran A, Oggero R (2005). Z-score of weight for age of infants with atopic dermatitis and cow’s milk allergy fed with a rice-hydrolysate formula during the first two years of life. Acta Paediatr Suppl.

[32] Agostoni C, Fiocchi A, Riva E, Terracciano L, Sarratud T, Martelli A (2007). Growth of infants with IgE-mediated cow’s milk allergy fed different formulas in the complementary feeding period. Pediatr Allergy Immunol.

[33] de Jong NW, Sprikkelman AB, Oude Elberink HN, Arends NJ, Vlieg-Boerstra BJ (2014). Blinded sensory evaluation of extensively hydrolyzed formulas and amino acid formulas. Ann Allergy Asthma Immunol.

[34] Miraglia Del Giudice M, D’Auria E, Peroni D, Palazzo S, Radaelli G, Comberiati P (2015). Flavor, relative palatability and components of cow’s milk hydrolysed formulas and amino acid-based formula. Ital J Pediatr.

[35] Medjad-Guillou N, Henocq A, Arnaud-Battandier F (1992). [Does the hydrolysis of proteins change the acceptability and the digestive tolerance of milk for infants? The results of a comparative and randomized prospective study]. Ann Pediatr (Paris).

[36] Schulz KF, Altman DG, Moher D, CONSORT Group (2010). CONSORT 2010 Statement: updated guidelines for reporting parallel group randomised trials. BMC Med.

