# Supplementary material for: Tolerance and growth in children with cow’s milk allergy fed a thickened extensively hydrolyzed casein-based formula
Source: BMC Pediatr. 2016 Jul 18;16:96. doi: 10.1186/s12887-016-0637-3 (PMC4950604; doi:10.1186/s12887-016-0637-3)
Supplement: Additional file 1: Table S1. — Supplementary baseline characteristics of FAS population (N = 30) at inclusion. (DOCX 16 kb) [file 12887_2016_637_MOESM1_ESM.docx]

**Additional file: Table S1.** Supplementary baseline characteristics of FAS population (N=30) at inclusion.

| Characteristics | | |
| --- | --- | --- |
| Number of live-born infants from the same pregnancy, N (%) | | |
| 1 | | 28 (93.3) |
| 2 | | 2 (6.7) |
| Number of siblings, N (%) | | |
| 0 | | 18 (60.0) |
| 1 | | 10 (33.3) |
| 2 | | 2 (6.7) |
| Mother education level, N (%) | | |
| Basic minimum education | | 2 (6.9) |
| Additional education but not to tertiary level | | 17 (58.6) |
| Tertiary education | | 10 (34.5) |
|  | Mother | Father |
| Anthropometric data of parents at study recruitment | | |
| Weight, mean( ± SD), kg | 66.3 (13.7) | 80.8 (11.2) |
| Height, mean (± SD), cm | 160.9 (6.9) | 179.4 (6.4) |
| Body mass index, mean (± SD), kg/m² | 25.8 (5.0) | 25.1 (2.8) |
| Socioeconomic status of parents, N (%) | | |
| Higher managerial / professional | 6 (20.0) | 5 (16.7) |
| Intermediate managerial / technical | 5 (16.7) | 6 (20.0) |
| Skilled (non-manual) | 6 (20.0) | 6 (20.0) |
| Skilled (manual) | 4 (13.3) | 9 (30.0) |
| Semi and unskilled manual workers | 2 (6.7) | 3 (10.0) |
| No professional activity | 7 (23.3) | 1 (3.3) |

N: Number of subjects.
